# Organic Acids in Rabbit Nutrition: Mechanisms, Advancements, and Potentials for Sustainable Production

**DOI:** 10.3390/vetsci13070620

**Published:** 2026-06-26

**Authors:** Tarek A. Ebeid, Mohamed Tharwat, Sohail Ahmad, Ahmed O. Abbas, Abdullah N. Alkhalaf, Fahad A. Alshanbari

**Affiliations:** 1 Department of Animal and Poultry Production, College of Agriculture and Food, Qassim University, Buraydah 51452, Saudi Arabia; t.ebeid@qu.edu.sa; 2 Department of Clinical Sciences, College of Veterinary Medicine, Qassim University, Buraidah 51452, Saudi Arabia; atieh@qu.edu.sa; 3 Department of Poultry Production, Faculty of Animal Production and Technology, University of Veterinary and Animal Sciences, Lahore 54000, Pakistan; sohail.ahmad@uvas.edu.pk; 4 Department of Animal and Fish Production, College of Agricultural and Food Sciences, King Faisal University, Al-Hofuf 31982, Saudi Arabia; aabbas@kfu.edu.sa; 5 Department of Pathology & Laboratory Diagnosis, College of Veterinary Medicine, Qassim University, Buraydah 51452, Saudi Arabia; akhlf@qu.edu.sa; 6 Department of Medical Biosciences, College of Veterinary Medicine, Qassim University, Buraidah 51452, Saudi Arabia

**Keywords:** antioxidant status, gut health, immunity, intestinal histomorphology, microbiota, organic acids, rabbits

## Abstract

Sustainable rabbit production requires effective nutritional strategies to enhance productivity, health status, and immune competence. Following the restriction of antibiotic growth promoters, organic acids (OAs) have gotten increasing attention as promising functional feed additives due to their multiple biological roles. This review aims to offer a comprehensive overview of the functional roles of OAs in rabbit nutrition, with a focus on their effects on gut morphology, nutrient digestibility, intestinal microbiota, antioxidative status, immunity, and growth performance in growing rabbits. The OAs may modulate gut microbiota balance through inhibition of pathogenic bacteria and promotion of beneficial microbial populations, thereby contributing to the establishment of a balanced intestinal ecosystem. This effect is particularly important during the post-weaning period, a critical stage characterized by increased susceptibility to enteric disorders and associated economic losses. The OAs may also enhance digestive enzyme activities, leading to improving nutrient digestibility, feed efficiency, and reducing feed wastage. In addition, OAs have been shown to improve intestinal histomorphology through coordinated effects on epithelial proliferation, mucosal renewal, and tight junction integrity. Furthermore, OAs have been shown to modulate antioxidative status and immune responses, which are essential for maintaining intestinal health and overall production sustainability. Collectively, OAs represent a promising and viable nutritional strategy to enhance the sustainability and efficiency of rabbit production systems through their beneficial effects on gut health, nutrient utilization, immune competence, and antioxidative status.

## Simple Summary

The use of antibiotics as growth promoters in rabbit production has been increasingly limited, creating a need for safe and effective alternatives. Organic acids are broadly used in animal nutrition and have shown potential to improve rabbit health and productivity. They help maintain a balanced gut microbiota by reducing harmful bacteria and supporting beneficial ones, particularly during the sensitive post-weaning period. Organic acids may also enhance digestion, strengthen the intestinal barrier, and support immune and antioxidant functions. However, their effectiveness could vary depending on factors such as type, dose, and method of application.

## 1. Introduction

Rabbits (*Oryctolagus cuniculus*) are herbivorous hindgut fermenters, and they rely on microbial fermentation in the cecum and large intestine to break down tough plant fibers [1]. In rabbits, the cecum serves as a large anaerobic fermentation chamber containing metabolically active microbiota that contributes to the breakdown of “ferments” (insoluble polysaccharides like cellulose and hemicellulose) into volatile fatty acids (VFAs) and utilizes ammonia derived from dietary protein and endogenous nitrogen metabolism for microbial protein synthesis [2]. This anaerobic fermentation process produces substantial amounts of VFAs, mainly acetate, propionate, and butyrate. These acids are absorbed through the walls of the colon and the cecum and they serve as a crucial energy source and help maintain a healthy intestinal environment [3]. Therefore, the establishment and maintenance of a stable cecal microbial ecosystem are essential for efficient nutrient utilization, optimal fermentation activity, epithelial integrity, and resistance to enteric pathogens. Consequently, intestinal health and microbial stability are critical determinants of nutrient utilization, growth performance, feed efficiency, and survival in sustainable rabbit production systems. However, the abrupt dietary change at weaning, coupled with incomplete immune and digestive functions, often leads to a disruption of cecal microbial balance, making rabbits susceptible to intestinal disorders, including rabbit epidemic enteropathy and post-weaning diarrhea, which remain major causes of economic losses in commercial rabbit production [2,4].

Several nutritional strategies and functional feed additives that are able to stabilize gut microbiota, improve intestinal functionality, and reduce pathogen proliferation have gained much attention in recent years. Among these alternatives, organic acids (OAs) have received increasing attention as potential functional feed additives in rabbit nutrition owing to their reported effects on gut microbial balance, gastrointestinal physiology, nutrient utilization, and productive performance [5,6]. The OAs are weak carboxylic acids including mono-carboxylic acids (e.g., formic, acetic, propionic, and butyric acids) or carboxylic acids with the hydroxyl group (e.g., lactic, malic, tartaric, and citric acids) or short-chain carboxylic acids containing double bonds (e.g., fumaric and sorbic acids) [7]. Their antimicrobial activity is strongly influenced by their dissociation constant (pKa), which determines the proportion of dissociated and undissociated forms within the gastrointestinal tract (Table 1). The OAs with higher pKa values (which are weaker acids) are often more effective antimicrobials in certain environments because they remain largely undissociated. In acidic conditions (like the stomach), OAs remain predominantly in their undissociated form, allowing them to diffuse across microbial cell membranes. Once inside the bacterial cytoplasm, where pH is relatively neutral, the acids dissociate into protons (H^+^) and anions (A^−^), resulting in intracellular acidification, disruption of enzymatic activity, impairment of nutrient transport systems, and inhibition of bacterial growth [8]. This mechanism selectively suppresses acid-sensitive pathogenic bacteria such as *Escherichia coli*, *Salmonella*, and *Clostridium* spp. while favoring more acid-tolerant beneficial bacteria, including *Lactobacillus* and *Bifidobacterium* spp. [9,10].

**Figure 1:**
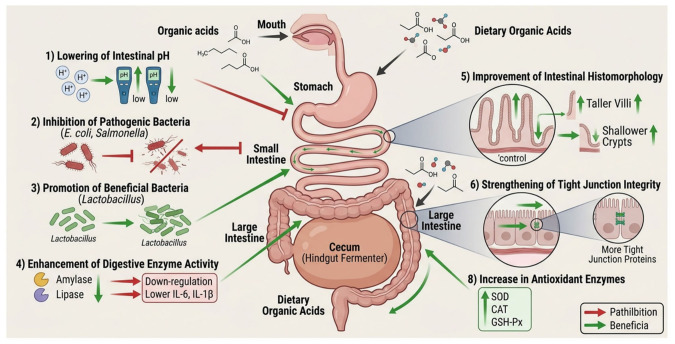
Mechanism of action of organic acids in the rabbit gut.

Beyond their antimicrobial properties, OAs exert broader physiological effects on intestinal functionality and host metabolism in rabbits. Dietary OAs may enhance intestinal morphology and stimulate digestive enzyme activity, thereby improving nutrient digestibility and feed efficiency in fattening rabbits [5,11,12,13]. Furthermore, OAs have been shown to improve antioxidative status, immune function, and inflammatory responses, which collectively contribute to the maintenance of intestinal barrier integrity [14,15] (Table 2). Such effects may be particularly important during the post-weaning period, when intestinal inflammation, oxidative stress, and microbial instability frequently compromise rabbit health and productive performance. Despite growing interest in the use of OAs in rabbit nutrition, their mechanisms of action and the consistency of their effects under different production conditions remain incompletely understood. Therefore, the aim of this review is to provide a comprehensive overview of the current knowledge regarding the effects of OAs on gut morphology, nutrient digestibility, intestinal microbiota, antioxidative status, immunity, and growth performance in growing rabbits (Figure 1).

## 2. Literature Search Strategy

The literature included in this review was retrieved through comprehensive searches of major scientific databases, including Web of Science, Scopus, PubMed, and Google Scholar. The search was conducted using combinations of the following keywords: rabbit, organic acids, acidifiers, citric acid, formic acid, propionic acid, butyric acid, sodium butyrate, gut health, intestinal morphology, nutrient digestibility, microbiota, immune response, antioxidant status, growth performance, carcass traits, and meat quality. Priority was given to studies published in peer-reviewed scientific journals between 2015 and 2025, although earlier seminal references were included when necessary to provide essential background information on rabbit digestive physiology and cecal fermentation. The review primarily focuses on studies conducted in rabbits; however, selected evidence from other monogastric species was included where relevant to explain biological mechanisms or to provide additional physiological context when rabbit-specific data were limited. Conference abstracts, non-peer-reviewed publications, and studies lacking sufficient methodological information were excluded whenever possible.

## 3. Impact of Organic Acids on Intestinal Histomorphometry

The intestinal morphology is a key determinant of digestive efficiency, nutrient absorption capacity, and mucosal barrier integrity, all of which ultimately influence growth performance in rabbits. This is particularly important during the post-weaning period, when animals are highly susceptible to enteric disorders [18]. The intestinal mucosa structure such as the villus height, crypt depth, ratio of villus height/crypt depth, mucosal thickness, and goblet cell density have effects on the absorptive surface area, turnover of epithelial cells, and immunity [19]. Several studies indicated that dietary OAs, particularly short-chain fatty acids (SCFAs) such as butyric and acetic acids, had positive effects on intestinal architecture such as raising the height of the villus, reducing the depth of the crypt, and raising the ratio of villus height to crypt depth in growing rabbits [6,15,16,20]. These results suggest that dietary OAs enhanced the absorptive and protective capacity of the gastrointestinal tract, particularly during the critical post-weaning period. From a physiological perspective, increased villus height expands the absorptive surface area, whereas reduced crypt depth reflects lower epithelial turnover and reduced intestinal stress, leading to enhancing gut physiology and health. A study mentioned that dietary supplementation with 0.5% sodium butyrate enhanced ileal villus height, reduced jejunal crypt depth, and improved the villus-height-to-crypt depth ratio in growing rabbits [16]. Similarly, dietary supplementation with coated sodium butyrate (250 g/ton feed) improved duodenal morphometry, including villus height, villus width, and duodenal length [20]. Another study reported that dietary sodium butyrate (0.75 g/kg diet) improved the integrity of the villi and number of goblet cells in the cecum of growing rabbits [6]. These results suggest that butyrate may directly support intestinal epithelial energy metabolism, thereby contributing to villus development, epithelial proliferation, and goblet cell differentiation. However, the precise mechanisms underlying these responses in rabbits require further investigation. Recent studies reported that dietary sodium acetate (2 g/kg diet) and sodium butyrate (0.75 g/kg diet) enhanced enterocyte proliferation and migration via activation of the Wnt/β-catenin signaling pathway in growing rabbits [6,15]. In addition, dietary sodium butyrate (0.75 g/kg) upregulated the expression of cell cycle- and apoptosis-related genes (*BCL*, *CASP3*, and *BAX*) in cecal tissue of New Zealand White (NZW) growing rabbits, indicating a regulatory role of OAs in epithelial renewal and in maintaining the balance between proliferation and programmed cell death, thereby preserving mucosal homeostasis and intestinal barrier integrity [6]. In addition, dietary inclusion of citric acid (1.0–2.0%) improved villus height and the villus-height-to-crypt depth ratio in the ileum of broiler rabbits [6,11,12]. Likewise, dietary citric acid (10 g/kg diet) was reported to slightly improve intestinal tissue architecture in the mucosal and submucosal layers of NZW growing rabbits [21]. Furthermore, dietary supplementation with a mixture of formic and citric acids [0.4% during the post-weaning period (28–55 d) and 0.2% during the finishing period (56–77 d)] increased villus height in the jejunum of growing rabbits [22]. Molecularly, dietary supplementation with 1000 mg/kg of mixed OAs (formic acid and propionic acid) increased the expression of Zonula occludens-1 (ZO-1) in the jejunal mucosa of Ira rabbits, a key protein involved in tight junction integrity and regulation of paracellular permeability [14]. It was reported that a combination of butyric acid ester, medium-chain fatty acids, and prebiotic *Saccharomyces cerevisiae* yeast cell walls (1 g/kg diet) increased villus height and reduced crypt depth in the duodenum of growing rabbits [5]. These improvements may be partly explained by synergism between OAs and other functional feed additives, such as medium-chain fatty acids and prebiotics, which may synergistically enhance gut health. Moreover, OAs serve as an energy source in the intestine through their involvement in the citric acid cycle and energy metabolism, thereby supporting tissue maintenance and development [23]. In addition, fumaric and citric acids participate in the citric acid cycle, while butyric acid acts as a direct energy source for enterocytes, thereby promoting villus growth and epithelial turnover [24].

**Figure 2:**
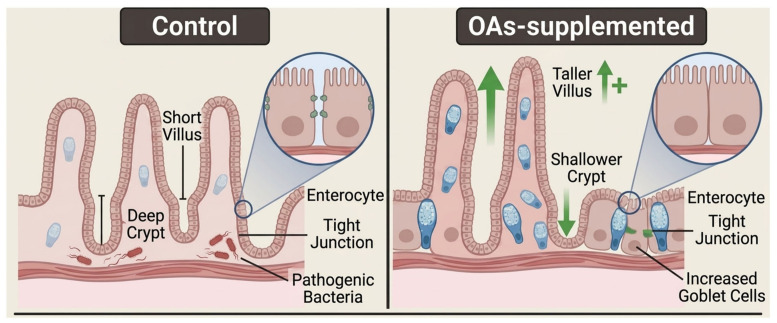
Impact of organic acids on intestinal histomorphology (control vs. OAs-supplemented). Green arrows indicate increased villus height, reduced crypt depth, improved villus-to-crypt ratio, and enhanced tight junction integrity.

Regarding intestinal physical dimensions, several studies have also suggested that dietary OAs positively influence intestinal length and weight [5,25]. For instance, dietary supplementation with butyric acid, citric acid, calcium formate, calcium propionate, and silicic acid (2 g/kg diet) increased the intestinal length by 1% and the intestinal weight by 5% in growing rabbits [25]. It is also noteworthy that enterocytes can efficiently absorb dietary OAs and utilize them as an energy source to promote cell growth, differentiation, and proliferation in the small intestine [26]. Another important aspect emerging from these findings is the apparent interaction between OAs and gut microbiota. By lowering intestinal pH and suppressing pathogenic bacteria, OAs create a more favorable luminal environment for beneficial bacteria, leading to reducing inflammation and enhancing intestinal cell growth and development. This concept is supported by evidence in monogastric animals such as rabbits [11,17], poultry [27], and weaned piglets [10], suggesting interactions between OAs, gut microbial communities, and the intestine. Taken together, dietary OAs may improve intestinal histomorphology through integrated effects on epithelial proliferation, mucosal renewal, and tight junction integrity in growing rabbits. These effects are essential for improving gut functionality and supporting sustainable rabbit production (Figure 2).

## 4. Impact of Organic Acids on Nutrients’ Digestibility

Rabbits are delicate hindgut fermenters; therefore, nutrient digestibility is a key determinant of growth performance, feed efficiency, and gastrointestinal health, particularly in reducing the risk of enteric disorders such as enteritis. Several studies have reported that dietary OAs improved the apparent digestibility of nutrients in growing rabbits [12,28,29]. For instance, dietary supplementation with 2.0% citric acid increased the digestibility of dry matter (DM), crude protein (CP), and ether extract (EE), while no significant effects were observed for crude fiber (CF) and nitrogen-free extract (NFE) in growing rabbits [28]. Similarly, Maklad et al. [12] reported that citric acid supplementation (0.5–1.0%) improved EE digestibility and the ratio of digestible energy intake to digestible CP intake, whereas NFE digestibility and total digestible nutrients (TDNs) remained unaffected. In another study, the inclusion of citric acid (1.0–1.5%) improved the digestibility coefficients of EE and NFE and also enhanced CP, CF, and EE digestibility in growing rabbits [29]. These findings elucidated that dietary citric acid improved CP and EE digestibility, which may have important physiological implications for protein accretion and energy utilization. Furthermore, another study indicated that dietary supplementation of 0.025% acetic acid or 0.05% propionic acid resulted in numerical improvements in the digestibility of CP, CF, and NFE in NZW growing rabbits [30]. These improvements in nutrient digestibility may be partly attributed to enhanced digestive enzymes’ activities, improved intestinal histomorphology, and a stabilized intestinal microbial ecosystem. In support of this, dietary acetic acid (1.5%) has been shown to increase the activities of amylase, lipase, and protease in growing rabbits, thereby improving the digestion of carbohydrates, lipids, and proteins, respectively [31]. In addition, OAs may lower gastric pH, thereby facilitating the conversion of pepsinogen to active pepsin and enhancing protein digestion [13,17]. Furthermore, reduced gut pH may also stimulate secretin release, which in turn promotes endogenous pancreatic enzyme secretion [6,13,32]. This mechanism is particularly important in rabbits because efficient enzymatic digestion in the foregut reduces the flow of undigested nutrients into the hindgut, thereby stabilizing cecal fermentation and reducing the risk of digestive disorders. Collectively, these mechanisms suggest that OAs may improve digestive efficiency through combined effects on enzyme activity, gastric acidity, and intestinal morphology.

Nevertheless, contrasting findings have also been reported. Several studies indicated that citric acid supplementation has limited or no significant effects on nutrient digestibility, except for CP in some cases [11,33]. For example, inclusion of 1.0% citric acid did not significantly affect DM, CF, EE, and NFE digestibility, although CP digestibility showed improvement [11]. Similarly, supplementation with 2% citric acid had no significant effects on DM, CF, EE, and NFE digestibility, with only a non-significant increase in CP digestibility [33]. In addition, dietary citric acid (10 g/kg diet) did not influence the digestibility of DM, CP, CF, EE, ash, or NFE in broiler rabbits [21]. One possible explanation of the absence of consistent responses may be explained by the lack of measurable effects of OAs on digestive enzyme activities in some physiological and dietary conditions [11,14]. Indeed, some studies indicated that dietary citric acid (1.0%) did not significantly affect amylase, cellulase, or trypsin activities in NZW growing rabbits [11], and supplementation with 1000–2000 mg/kg of an OA mixture (formic acid and propionic acid) did not alter α-amylase, trypsin, or lipase activity in jejunal mucosa of Ira rabbits [14]. Such findings suggest that the level of acidification attained may not consistently be adequate to effect changes in digestive secretions or intestinal functionality to a biologically significant degree. Overall, the variability in responses suggests that the effects of OAs on nutrient digestibility are highly dependent on several interacting factors, including dosage, acid type, dietary composition, animal age, health status, and environmental conditions. In particular, the buffering capacity of the diet may neutralize the acidifying effect of OAs, thereby limiting their potential benefits on digestive processes and nutrient utilization. Furthermore, rabbits raised in optimal hygienic and nutritional conditions may demonstrate weaker responses, as their baseline digestive efficiency is already high. In contrast, animals exposed to post-weaning stress or microbial challenges may exhibit more pronounced reactions.

## 5. Impact of Organic Acids on Intestinal Microbiota and Health

**Figure 3:**
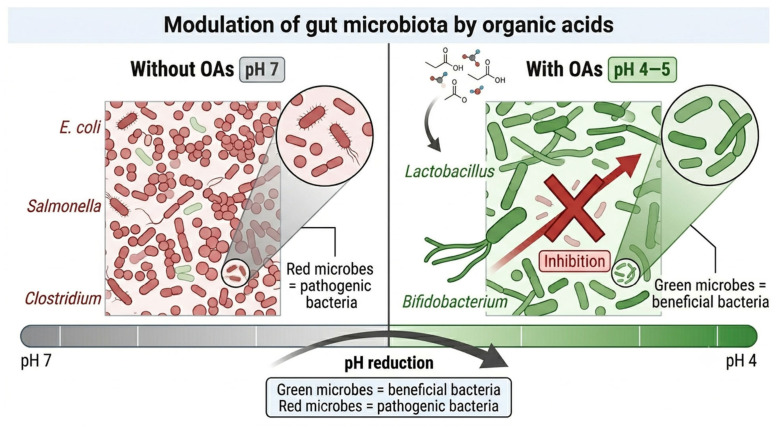
Modulation of gut microbiota by organic acids. Green microbes represent beneficial bacteria (e.g., *Lactobacillus*, *Bifidobacterium*). Red microbes represent pathogenic bacteria (e.g., *E. coli*, *Salmonella*). Red arrow indicates inhibition. Bottom pH scale shows acidification effect.

The gut microbiota plays a central role in rabbit production efficiency, as rabbits are hindgut fermenters in which the cecum represents the primary site for microbial fermentation, short-chain fatty acid (SCFA) production, and pathogen exclusion [18]. Therefore, the establishment and maintenance of a stable and balanced gut microbiome are critical for rabbits, especially during the post-weaning vulnerable period, to avoid gastrointestinal diseases and support digestive efficiency, immune competence, and overall health status [34]. A growing body of evidence indicates that dietary OAs played a beneficial role in maintaining a balance in the microbiome by preventing the growth and proliferation of pathogenic bacteria in the gut and enhancing the beneficial bacteria in the gut in growing rabbits [11,17,31,35]. It is important to note that the majority of studies investigating the effects of OAs on the rabbit gut microbiota have employed culture-dependent methods or targeted analyses of specific bacterial populations, primarily *Lactobacillus*, *E. coli*, and *Salmonella*. For example, supplementation with an OA mixture (ammonium formate, acetic acid, and formic acid) in drinking water at different concentrations (0.55 g/kg—pH 5, 0.85 g/kg—pH 4.3, and 3.3 g/kg—pH 3.6) decreased the *E. coli* count, decreased the relative proportions of *E. coli* to *Bacteroides-Prevotella* and to total count, increased the *Lactobacillus* count, and increased the relative proportion of *Lactobacillus* to total count in cecum of growing rabbits [17]. Similarly, another study found that dietary inclusion of 1.5% acetic acid reduced the counts of *E. coli* and *Salmonella* in both ileum and cecum [31]. Moreover, it was reported that supplementation with 1.0% citric acid improved cecal microbial balance by increasing *Lactobacillus* and decreasing *E. coli* populations in NZW growing rabbits [11]. These findings indicated that dietary OAs contribute to intestinal microbial community stabilization in growing rabbits. These microbiota shifts are largely attributed to the acidifying effect of OAs on the gastrointestinal environment and reducing pH values [6,15,25,35]. In this context, dietary sodium butyrate (0.75 g/kg diet) or sodium acetate (2 g/kg diet) reduced pH in the duodenum and cecum and modified SCFA profiles in NZW growing rabbits [6,15]. These findings indicate that low duodenal and cecal pH creates an unfavorable environment for acid-sensitive pathogenic bacteria, while selectively favoring acid-tolerant beneficial bacteria. Likewise, drinking water supplementation with OAs (acetic, formic, propionic, lactic, citric, or butyric acids) at different pH levels (3, 4, and 5) significantly decreased ileal and cecal pH [13]. In agreement, dietary inclusion of a mixture of butyric acid, citric acid, calcium formate, calcium propionate, and silicic acid (2 g/kg diet) also reduced pH across different intestinal segments [25], while 1.5% acetic acid supplementation significantly decreased ileal and cecal pH [31]. Mechanistically, a low gastrointestinal pH environment inhibits acid-sensitive pathogenic bacteria such as *Salmonella*, *Clostridium*, and *E. coli*, while favoring acid-tolerant beneficial genera including *Lactobacillus*, *Bacillus*, and *Bifidobacterium* [9,10]. In addition, OAs exhibit both bacteriostatic and bactericidal effects [36]. The undissociated OAs are lipophilic, allowing them to enter the microbial cell. Once inside, they dissociate to protons (H^+^) and anions (A^−^), which results in the lowering of pH and affects normal cell functions, causing cell death [8]. Beyond pH-dependent effects, sodium butyrate has also been shown to modulate bacterial virulence gene expression, thereby reducing bacterial adhesion to the intestinal mucosa, leading to reducing bacterial pathogenicity [37]. Taken together, it could be concluded that OAs may have direct (pH-dependent) and indirect (pH-independent) antimicrobial and microbiota-modulating effects in increasing the abundance of beneficial bacteria and decreasing the abundance of opportunistic pathogenic bacteria. However, their efficacy may vary according to the specific acid used. For instance, formic and citric acids primarily act through acidification of the gastrointestinal environment, whereas butyrate may additionally influence bacterial virulence and host–microbiota interactions. These effects appear particularly beneficial during the post-weaning period, when rabbits are highly susceptible to microbial dysbiosis and enteric disorders (Figure 3). Nevertheless, comparative studies evaluating the specific effects and mechanisms of individual OAs in rabbits remain limited. It should be noted that most of the available studies evaluating the effects of OAs on rabbit gut microbiota have relied on culture-dependent techniques or targeted quantification of selected bacterial groups, particularly *Lactobacillus*, *E. coli*, and *Salmonella*. While these approaches provide valuable information regarding specific microbial populations, they capture only a limited proportion of the highly diverse and complex cecal microbial ecosystem. Many intestinal microorganisms are difficult or impossible to cultivate under conventional laboratory conditions, which may result in an incomplete characterization of microbial responses to OA supplementation. Therefore, further studies integrating high-throughput microbiome sequencing, metagenomics, metabolomics, and intestinal transcriptomics are required to elucidate the mechanisms underlying OA-mediated modulation of the gut ecosystem and to optimize OA-based nutritional strategies under commercial rabbit production conditions.

## 6. Impact of Organic Acids on Antioxidative Properties

Oxidative status is a key determinant of rabbit health and productivity, as excessive generation of reactive oxygen species (ROS) can impair intestinal integrity, compromise immune function, and reduce growth performance [38]. Oxidative stress occurs when ROS production exceeds the capacity of endogenous antioxidant defense systems, leading to oxidative damage to lipids, proteins, and DNA [39]. In this context, antioxidant enzymes such as catalase (CAT), glutathione peroxidase (GSH-Px), and superoxide dismutase (SOD) play a central role in neutralizing reactive intermediates and maintaining cellular redox balance [38]. Modern rabbits are highly susceptible to oxidative stress due to their rapid growth rate, high metabolic activity, and sensitivity to environmental and nutritional stressors [40]. Thus, maintenance of redox homeostasis is essential for sustainable rabbit production. Interestingly, a growing number of studies have demonstrated that dietary OAs can positively modulate antioxidant status in rabbits [6,11,14,15]. For instance, dietary supplementation with 2000 mg/kg of a mixed OA preparation (formic acid and propionic acid) increased hepatic CAT activity and total antioxidant capacity (T-AOC) while reducing hepatic malondialdehyde (MDA, a marker of lipid peroxidation) in Ira rabbits (*p* < 0.05). However, hepatic SOD, GSH-Px, glutathione (GSH), oxidized glutathione (GSSG), and the GSH/GSSG ratio were not significantly affected at either 1000 or 2000 mg/kg inclusion levels in Ira rabbits [14]. Reduction in MDA concentrations further supports the protective role of OAs against lipid peroxidation and cell membrane damage. Similarly, dietary supplementation with 1% citric acid increased plasma SOD activity, whereas plasma MDA and GSH-Px levels remained unchanged, indicating a moderate and selective improvement in antioxidant status [11]. These positive findings indicate that these antioxidant enzymes may be involved in detoxification and scavenging free radicals, leading to enhanced cellular protection and physiological stability in rabbit production systems. In addition, more consistent effects have been observed in gut-associated antioxidant responses. Dietary sodium butyrate supplementation (0.75 g/kg diet) increased cecal antioxidant enzyme activities, including SOD and GSH-Px, in NZW growing rabbits, suggesting improved protection against oxidative stress at the intestinal level [6]. Likewise, dietary sodium acetate (2 g/kg) enhanced cecal antioxidant capacity by increasing T-AOC, SOD, and GSH-Px activities without altering MDA levels [15]. These findings indicate that the antioxidative effects of OAs may be more pronounced in the gastrointestinal tract than in systemic circulation. Such benefits are important to preserve the intestinal mucosa of oxidants, microbial toxins, free radicals, and inflammatory stimuli. At the molecular level, the mechanisms underlying the antioxidant effects of OAs in rabbits remain incompletely understood. Although dietary OAs have been shown to enhance antioxidant enzyme activities and improve redox status in rabbits, the intracellular pathways mediating these responses have not been fully elucidated. Evidence from studies in other animal species and experimental models suggests that the antioxidant potential of certain OAs (e.g., propionic acid) has been associated with activation of the Keap1/Nrf2 signaling pathway, which promotes nuclear translocation of Nrf2 and subsequent binding to antioxidant response elements, thereby upregulating the expression of endogenous antioxidant enzymes [41]. In addition, formic and citric acids have been reported to modulate cellular redox status by influencing thiol metabolism and increasing reduced glutathione levels [42]. Since glutathione plays a pivotal role in detoxification, free radical scavenging, and maintaining mitochondrial function, modulation of glutathione metabolism may represent another important mechanism underlying the protective effects of OAs. However, these mechanisms have not been directly demonstrated in rabbits and should therefore be considered as potential pathways that warrant further investigation. Overall, available evidence suggests that OAs may enhance antioxidant defense in rabbits primarily through stimulation of endogenous antioxidant enzymes and modulation of redox-regulating signaling pathways such as Keap1/Nrf2. However, the reported responses are not entirely consistent across studies, as effects vary according to the type of OA, inclusion level, tissue examined, and antioxidant biomarker evaluated. These effects may contribute to improving intestinal epithelial integrity and overall health status. Consequently, the antioxidative role of OAs represents an important mechanism supporting sustainable rabbit production systems. It should be noted that several recent findings regarding the effects of OAs on antioxidative status in rabbits are derived from a relatively limited number of studies. Therefore, further independent investigations are required to validate these observations under different production systems and experimental conditions.

## 7. Impact of Organic Acids on Immunity

Immune system integrity and efficiency play a critical role in rabbit health and productivity in especially intensive production systems [43]. In the gastrointestinal tract, immunity is maintained by a complex network comprising the mucosal immune barrier, gut-associated lymphoid tissue, immune cells (B and T lymphocytes), immunoglobulins, and cytokines, all of which collectively contribute to pathogen exclusion and intestinal homeostasis [44]. Dietary OAs have been shown to modulate both local and systemic immune responses in rabbits through multiple mechanisms [14,16]. For example, supplementation with mixed OAs (1000 mg/kg, formic acid and propionic acid) increased jejunal secretory immunoglobulin A (sIgA) and anti-inflammatory cytokine interleukin-10 (IL-10) concentrations while downregulating the gene expression of pro-inflammatory cytokines IL-6 and IL-1β in Ira rabbits (*p* < 0.05) [14]. Secretory IgA is the first immunological barrier at the intestinal surface, which limits pathogen adhesion, neutralizes enteric toxins, maintains the microbiome balance, and regulates immune cells residing in or attracted to mucosal tissues. Therefore, increased jejunal sIgA concentrations due to dietary OA supplementation indicate improved mucosal immune competence. Additionally, the modulation of cytokine profiles by OAs further highlights their anti-inflammatory potential. Additionally, dietary sodium butyrate (0.5%) enhanced intestinal immune function by increasing sIgA, IL-4, and interferon-γ (IFN-γ) levels across different intestinal segments in growing rabbits, indicating improved mucosal immune competence [16]. This immune enhancement was also associated with increased expression of the tight junction protein occludin, suggesting a link between immune modulation and barrier integrity [16]. The integrity interaction among immune competence, antioxidant status, microbial balance, and epithelial function highlights the multifactorial nature of OA activity in rabbits. At the cellular level, dietary sodium acetate (2 g/kg diet) or sodium butyrate (0.75 g/kg diet) increased the activity of Na^+^-K^+^ ATPase and Ca^2+^-Mg^2+^ ATPase in the cecum of NZW growing rabbits [15]. Although these enzymes are not direct indicators of immune function, they are essential for maintaining ion transport, membrane integrity, and cellular homeostasis. Consequently, the observed increases may reflect improved intestinal epithelial functionality and physiological status, which could indirectly support mucosal barrier function, immunity, and intestinal health. In agreement, studies in other monogastric species have demonstrated that OAs can suppress inflammatory signaling pathways; for instance, glyceryl butyrate reduced IL-1β, IL-6, and TNF-α expression in weaned piglets by inhibiting NF-κB/MAPK signaling [45]. Likewise, sodium butyrate has been reported to enhance goblet cell activity and mucin gene expression, thereby strengthening mucosal barrier defenses in broiler chickens [46,47]. In addition to innate immunity, OAs also appear to influence systemic immune responses in rabbits [11,14,28]. Dietary citric acid (1%) increased plasma concentrations of immunoglobulins IgM and IgG in growing rabbits [11]. In another study, a linear increase in the number of lymphocytes was observed with the increasing concentration of dietary citric acid (0.5, 1.0, 1.5, 2.0, and 2.5%) [28]. These findings suggest that citric acid may modulate certain immune-related parameters in rabbits. However, the biological significance of increased immunoglobulin concentrations and lymphocyte counts should be interpreted with caution, as such responses may reflect immune activation or modulation rather than a direct enhancement of immune competence. Furthermore, supplementation with mixed OAs (1000–2000 mg/kg, formic acid and propionic acid) reduced serum IL-6 concentrations, indicating an anti-inflammatory systemic effect in Ira rabbits [14].

However, these conclusions are based primarily on indirect biomarkers, and their biological significance in terms of functional immune competence remains incompletely understood. Therefore, while OAs appear to influence immune and barrier-related responses, further studies incorporating direct assessments of immune function and disease resistance are required to clarify their immunological benefits in rabbits. Overall, it is speculated that dietary OAs may modulate several immune-related parameters in rabbits by strengthening mucosal immunity, increasing immunoglobulin production, modulating cytokine profiles toward an anti-inflammatory state, and improving epithelial barrier integrity. However, these conclusions are based primarily on indirect biomarkers, and their biological significance in terms of functional immune competence remains incompletely understood. Therefore, further studies are still required to clarify their effects on both humoral and cell-mediated immune responses under different physiological and management conditions in rabbits. Also, future studies of cellular signaling pathways are still needed to clarify the mechanisms underlying the role of OAs in immune modulation.

## 8. Impact of Organic Acids on Carcass and Meat Quality Characteristics

Carcass and meat quality traits are important determinants of profitability and consumer acceptability in rabbit production systems. It should be noted that the available evidence regarding the effects of OAs on carcass characteristics and meat quality in rabbits is derived from a relatively limited number of studies. Also, the available results of the effects of dietary OAs on carcass characteristics in rabbits remain inconsistent. Some studies have reported beneficial effects [30,33]. For example, dietary supplementation with 0.025% acetic acid or 0.05% propionic acid increased hot carcass yield and fur percentage, while 0.05% acetic acid increased cold carcass yield in NZW growing rabbits [30]. They also mentioned that OA supplementation (0.025% acetic acid, 0.05% acetic acid, or 0.025% propionic acid) reduced kidney fat percentage and increased heart and liver percentages [30]. Similarly, dietary inclusion of 2% citric acid resulted in a numerical increase in carcass yield and a reduction in abdominal fat in growing rabbits [33]. Increased hot and cold carcass yields in the OA group may reflect improved nutrient digestibility and greater efficiency of nutrient partitioning toward lean tissue deposition than fat accumulation. Also, the reduction in kidney or abdominal fat might be attributed to the fact that OAs may influence lipid metabolism and energy utilization. In contrast, numerous studies have reported no significant effects of OAs on carcass traits [5,11,21,48]. Dietary citric acid (10 g/kg diet) did not affect dressing percentage or relative weights of liver, heart, and kidney in NZW rabbits [21]. Likewise, supplementation with 1% citric acid showed no significant effects on carcass yield or organ weights, including liver, kidney, heart, lungs, giblets, and total edible parts [11]. Similarly, other reports indicated negligible effects of OAs on carcass characteristics [5,48]. In addition, dietary sodium acetate (2 g/kg diet) reduced liver, lung, and kidney weights but increased stomach weight and intestinal length in NZW rabbits [15], while sodium butyrate (0.75 g/kg diet) decreased heart, liver, and lung weights and increased cecal length [6]. The biological significance of these changes remains unclear, as no accompanying pathological or performance impairments were reported. Increased intestinal or cecal length may reflect adaptive responses associated with enhanced digestive and fermentative capacity, whereas the observed reductions in organ weights should be interpreted with caution. Therefore, these findings are currently best regarded as physiological modifications whose functional implications require further investigation. The absence of consistent responses suggests that carcass characteristics may be less sensitive to OA supplementation compared with intestinal and microbial parameters. Also, rabbit carcass composition of ES is influenced by multiple interacting factors including genetic factors, slaughter age, dietary energy density, environmental conditions, and growth rate, which may mask the relatively minor effects of OAs. Moreover, OA type, inclusion level, and dietary composition may further contribute to inconsistent outcomes among studies. Further studies are therefore required to determine optimal dosages and application strategies.

Regarding meat quality, the available literature is limited and presents conflicting results. Dietary sodium acetate (2 g/kg diet) improved meat chemical composition by increasing ash content and reducing triglyceride levels in the quadriceps femoris muscle of NZW rabbits, without affecting moisture, dry matter, or protein content. In addition, it positively influenced muscle fiber characteristics by increasing fiber density, total number, and total area while reducing fiber diameter [15]. Similarly, sodium butyrate (0.75 g/kg diet) increased muscle ash content but reduced concentrations of total amino acids, including essential amino acids and tyrosine [6]. This response may have implications for the nutritional quality of rabbit meat and contrasts with the generally positive effects of OAs reported on other production traits. However, as this finding is based on limited evidence, its biological significance and underlying mechanisms remain unclear and warrant further investigation. It also improved muscle fiber structure, characterized by more closely arranged fibers and increased fiber density and number, alongside reduced fiber diameter [6]. Increasing muscle ash content and reducing triglyceride concentration following sodium acetate supplementation may indicate enhanced mineral retention and altered lipid metabolism in broiler muscles. The observed modifications in muscle fiber characteristics are closely associated with meat tenderness, water-holding capacity, and textural properties. The observed modifications in muscle fiber characteristics may be relevant to meat quality, as muscle fiber morphology has been associated with technological and sensory attributes in meat-producing animals. However, because parameters such as tenderness, water-holding capacity, and textural properties were not directly evaluated in the cited studies, the implications of these structural changes for rabbit meat quality remain speculative and require further investigation. In contrast, dietary citric acid (10 g/kg diet) did not affect the chemical composition of rabbit muscle, including dry matter, crude protein, ether extract, and ash [21]. The inconsistency among studies may largely reflect differences in OA type, supplementation level, duration of feeding, dietary composition, slaughter age, and environmental conditions. In summary, current evidence regarding the effects of dietary OAs on carcass and meat quality in rabbits remains limited and inconsistent, preventing definitive conclusions. Therefore, further well-designed studies are needed to clarify their physiological mechanisms and to establish their practical implications for rabbit meat production.

## 9. Impact of Organic Acids on Growth Performance

**Figure 4:**
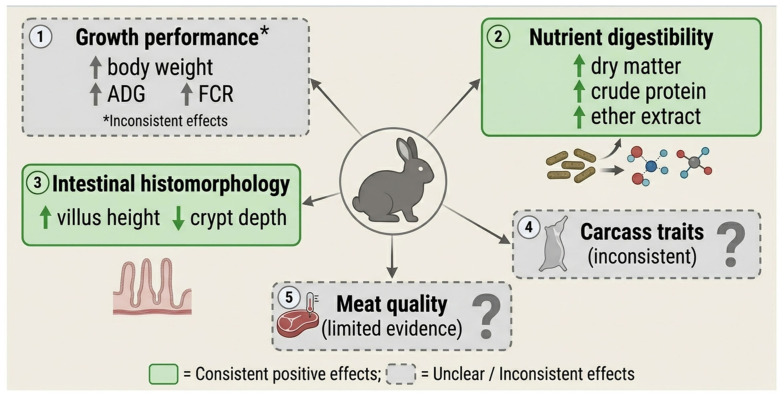
Summary of effects of organic acids on production parameters in rabbits (growth, digestibility, carcass, meat quality).

Growth performance is a key determinant of profitability and sustainability in rabbit production. However, the effects of OAs on growth performance indices, including body weight (BW), average daily gain (ADG), feed intake (FI), and feed conversion ratio (FCR), remain inconsistent in growing rabbits. Several studies have reported positive effects of dietary OAs on growth performance [5,6,15,17,20,25,28,29,30,33]. For instance, dietary supplementation with 0.025% acetic acid and 0.05% propionic acid improved live BW, ADG, FI, and FCR in NZW broiler rabbits [30]. Similarly, supplementation of an OA mixture (ammonium formate, acetic acid, and formic acid) in drinking water at different acidification levels (0.55 g/kg—pH 5, 0.85 g/kg—pH 4.3, and 3.3 g/kg—pH 3.6) enhanced ADG, FCR, and final BW in growing rabbits [17]. Moreover, dietary coated sodium butyrate (250 g/ton feed) increased BW, ADG, FI, and FCR [20]. These findings indicate that protected forms of butyrate may exert greater efficiency because they allow for the gradual release of distal intestinal fragments, thereby prolonging their antimicrobial actions along the gastrointestinal tract. These findings suggest that protected forms of butyrate may enhance butyrate delivery to distal intestinal segments through a gradual-release mechanism. Nevertheless, evidence demonstrating superior efficacy compared with unprotected butyrate in rabbits remains limited and requires further confirmation. In addition, supplementation with a combination of butyric acid ester, medium-chain fatty acids, and prebiotic *Saccharomyces cerevisiae* yeast cell walls (1 g/kg diet) improved growth performance and feed efficiency in growing rabbits [5]. These improvements suggest potential synergistic interactions between OAs and other functional feed additives, such as medium-chain fatty acids and prebiotics, in enhancing productivity. Furthermore, dietary inclusion of 2% citric acid improved final BW and ADG in growing rabbits [33]. It was reported that dietary supplementation of 1.5% fumaric acid increased ADG and FCR insignificantly [49,50]. Similar findings were posted by Hollister et al. [51]. Thus, it might be speculated that OAs may enhance growth performance through indirect improvements in intestinal morphology, nutrient digestibility, microbial balance, antioxidative status, and immune competence in growing rabbits. In contrast, various studies reported no significant effects of dietary OAs on growth performance in rabbits [11,14,16,21,22,52]. For example, a combination of formic acid and citric acid (0.4% during the post-weaning period: 28–55 d; 0.2% during the finishing period: 56–77 d) did not affect growth performance during the post-weaning phase, although ADG was higher during the finishing period compared with the control group (48.0 vs. 43.9 g), with no effect on mortality rate [22]. Similarly, sodium butyrate supplementation (0.5%) had no significant effect on ADG, FI, or FCR, although it reduced mortality and diarrhea incidence [16] (Li et al. 2020). These results may be partially supported by studies evaluating other acid-based feed additives. It was noted that dietary inclusion of 0.5% caprylic acid [53] or 1% triacylglycerols of caprylic and capric acid [54] reduced post-weaning mortality in growing rabbits. The reductions in diarrhea incidence and mortality rate might be associated with reducing the pathogenic load and improving gut health stability. Indeed, because caprylic and capric acids are generally classified as medium-chain fatty acids rather than conventional OAs, their effects should be interpreted separately from those of the OAs discussed in this review. In addition, dietary supplementation with 1% citric acid did not affect BW, FI, or FCR in growing rabbits [11], while 1000 mg/kg mixed OAs (formic acid and propionic acid) showed no effects on final BW, FI, FCR, or diarrhea rate in Ira rabbits [14]. Furthermore, the form of administration may influence responses. A study evaluating six OAs (acetic, formic, propionic, lactic, citric, and butyric acids) in drinking water at different acidification levels (pH 3, 4, and 5) reported that acetic, propionic, and butyric acids at pH 3 reduced water intake, FI, and ADG at 35 days of age. In contrast, higher BW and BW gains were observed in rabbits receiving formic and citric acids at pH 3 compared with other acids. Overall, rabbits receiving acidified water at pH 4 and 5 higher showed greater BW and BW gain than those receiving pH 3 water [22]. The lack of consistent responses suggests that the beneficial effects of OAs may depend on the degree of physiological or microbial challenge the animals face. Under optimal health and nutritional conditions, basal digestive efficiency may be sufficiently high to limit the observed effect of OA supplementation. Also, these responses may be associated with the organoleptic properties of OAs, particularly their strong taste and odor [55]. Collectively, these findings indicate that the efficacy of OAs on growth performance is strongly influenced by dose, acidification level, and administration form. Some studies have reported favorable responses with moderate dietary inclusion levels (0.5–1.0%) and moderate drinking water acidification (pH 4–5), whereas excessive acidification (e.g., around pH 3) has occasionally been associated with reduced feed and water intake, possibly due to decreased palatability. However, given the variability among studies, definitive recommendations regarding optimal inclusion rates and acidification levels cannot yet be established. Therefore, further research is required to determine optimal inclusion levels and combinations of OAs to maximize growth performance in rabbits (Figure 4).

## 10. Key Factors Influencing OAs’ Efficacy

**Figure 5:**
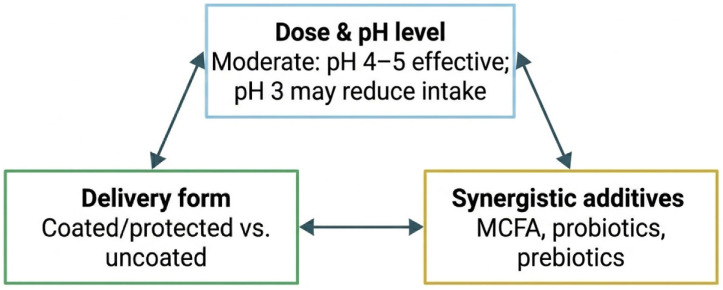
Factors influencing the efficacy of organic acids in rabbits. OA = organic acid; pH = acidity level; MCFA = medium-chain fatty acids.

The efficacy of OAs in rabbits appears to be influenced by multiple interacting factors, including acid type, inclusion level, administration route, diet composition, buffering capacity, animal age, health status, and environmental conditions. The variability in responses reported across studies suggests that no single factor can be identified as the primary determinant of OA effectiveness. In some studies, moderate acidification of drinking water, protected OA formulations, and combinations with other functional feed additives have been associated with beneficial outcomes (Figure 5). However, the evidence supporting these approaches remains limited and is not always consistent across experimental conditions. Therefore, such strategies should be regarded as promising rather than universally effective. Overall, the available literature indicates that the biological responses to OAs are highly context-dependent and influenced by complex interactions among dietary, physiological, and management factors. Despite the growing body of evidence, limited information is available regarding the effects of OAs on humoral- and cell-mediated immune responses as well as detailed meat quality characteristics in rabbits. In addition, the variability in reported results highlights the need for further research to determine optimal inclusion levels, appropriate chemical forms, and effective combinations of OAs to maximize their benefits in rabbit production systems.

## 11. Conclusions

Organic acids represent a promising and viable nutritional strategy to enhance the sustainability and efficiency of rabbit production systems through their beneficial effects on gut health, nutrient utilization, immune competence, and antioxidative status. Dietary supplementation with organic acids can modulate gastrointestinal pH, promote a balanced intestinal microbiota, improve intestinal histomorphology, enhance antioxidant defenses, and support immune function while reducing inflammation, thereby contributing to the maintenance of intestinal barrier integrity in growing rabbits. These effects are particularly relevant for the development of antibiotic-free and more sustainable rabbit production systems. However, the responses to organic acid supplementation are not always consistent across studies. While beneficial effects have frequently been reported for gut health-related traits, the effects on nutrient digestibility, growth performance, carcass characteristics, and meat quality are more variable and appear to depend on multiple interacting factors, including acid type, inclusion level, administration route, diet composition, animal age, health status, and management conditions. Furthermore, some conclusions regarding immune modulation, antioxidant mechanisms, and meat quality are based on a relatively limited evidence base and should therefore be interpreted with caution. Further well-designed studies are required to clarify their mechanisms of action, establish optimal application strategies, and determine the conditions under which consistent biological and productive responses can be achieved under commercial production systems.

## Figures and Tables

**Table 1:** Physicochemical properties of different organic acids.

Acid	pKa	Gross Energy (kJ/g)	Physical State	Solubility in Water
Formic acid	3.75	5.8	Liquid	High
Acetic acid	4.75	14.8	Liquid	High
Propionic acid	4.87	20.8	Liquid	High
Butyric acid	4.82	24.8	Liquid	High
Lactic acid	3.08	15.1	Liquid	Moderate
Malic acid	3.40/5.11	10.0	Solid	High
Citric acid	3.14/5.95/6.39	10.3	Solid	Moderate
Fumaric acid	3.03/4.44	11.5	Solid	Low
Sorbic acid	4.75	28.0	Solid	Low

**Table 2:** Summary of the impacts of organic acids on rabbits’ productivity and health.

Organic Acids	Level	Delivery Route	Physiological Category	Main Impacts	References
Sodium butyrate	0.75 g/kg diet	Feed	Gut development and health	Enhanced the integrity of epithelial villi and number of goblet cells in cecum. Stimulated enterocyte proliferation and migration via activating the Wnt/β-catenin signaling pathway Increased cell cycle- and apoptosis-related genes (*BCL*, *CASP3*, and *BAX*) in cecal tissue. Reduced duodenal and cecal pH.	[6]
			Oxidative status	Improved total superoxide dismutase and glutathione peroxidase activities in cecum.	
			Immune function	Enhanced activities of immune-related enzymes including Na^+^-K^+^ ATPase and Ca^2+^-Mg^2+^ ATPase in cecum.	
			Organ development	Decreased heart, liver, and lung weights. Increased cecum length.	
			Meat quality and muscle fiber characteristics	Increased crude ash content in meat. Reduced meat concentrations of tyrosine, delicious amino acids, and total amino acids. Improved muscle fiber characteristics including greater total number, bigger total area, greater density, and lower diameter.	
Sodium acetate	2 g/kg diet	Feed	Gut development and health	Stimulated enterocyte proliferation and migration via activating the Wnt/β-catenin signaling pathway. Decreased duodenal and cecal pH.	[15]
			Oxidative status	Increased total antioxidant capacity, total superoxide dismutase, and glutathione peroxidase activities in cecum. No significant effect on malondialdehyde levels in cecum.	
			Organ development	Decreased liver, lung, and kidney weights. Increased stomach weight and length of the small intestine and cecum.	
			Meat quality	Increased meat content of crude ash and decreased triglyceride content. No significant impact on moisture, dry matter, and protein concentrations in meat. Improved muscle fiber characteristics.	
Acetic, formic, propionic, lactic, citric, and butyric acids	pH 3, 4, and 5	Drinking water	Digestive function	Increased gastric pepsin activity. Reduced pH significantly in ileum and cecum.	[13]
			Growth performance	At pH 3, water intake, feed intake, water intake/feed intake ratio, and average daily gain were reduced. At pH 4 and pH 5, body weight gain and body weight at 35 days of age had improved.	
Citric acid	1.0%	Feed	Gut microbiota	Increased the *Lactobacillus* count and reduced the *E. coli* count.	[11]
			Oxidative status	Increased plasma superoxide dismutase activity, while malondialdehyde and glutathione peroxidase remained unchanged.	
			Immune function	Increased plasma concentration of immunoglobulins IgM and IgG.	
			Nutrient utilization	No significant influence on digestibility of dry matter, crude fiber, ether extract, and nitrogen-free extract, but enhanced crude protein digestibility. No significant effect on activities of amylase, cellulase, and trypsin.	
			Growth performance and carcass traits	No significant influence on live body weight, feed intake, and feed conversion ratio. No significant effect on carcass %, liver %, kidney %, heart %, lungs %, giblets %, and total edible parts %.	
Mixed organic acids (29% formic acid and 6% propionic acid)	1000–2000 mg/kg diet	Feed	Gut health and function	Enhanced gene expression of Zonula occludens-1 (ZO-1) in the jejunal mucosa. No significant impact on α-amylase, trypsin, and lipase in jejunal mucosa.	[14]
			Oxidative status	Enhanced hepatic catalase activity and total antioxidant capacity concentrations, while reduced hepatic malondialdehyde concentration.	
			Immune function	Increased jejunal secretory immunoglobulin A (sIgA) content and anti-inflammatory cytokine interleukin-10 concentration, while reduced gene expression of pro-inflammatory cytokines interleukin-6 and interleukin-1β. Decreased serum concentrations of interleukin-6.	
			Growth performance	No significant impact on final body weight, feed intake, feed conversion ratio, or diarrhea rate.	
Sodium butyrate	0.5%	Feed	Gut development	Increased ileal villus height, reduced jejunal crypt depth, and improved villus height/crypt depth ratio. Upregulation of occludin expression in duodenum.	[16]
			Immune function	Improved intestinal immune parameter secretory immunoglobulin A, interleukin-4, and interferon-γ in duodenum, jejunum, and ileum.	
			Growth performance	No significant influence on average daily gain, feed intake, and feed conversion ratio. Reduced mortality and diarrhea rates.	
Organic acid mixture (ammonium formate, acetic acid, and formic acid)	pH 3.6, 4.3, and 5.0	Drinking water	Gut microbiota	Reduced *E. coli* count reduced the relative proportions of *E. coli* to *Bacteroides*–*Prevotella* and to total count, increased *Lactobacillus* count, and incensed the relative proportion of *Lactobacillus* to total count.	[17]
			Digestive function	Increased gastric pepsin activity.	
			Growth performance	Enhanced average daily gain, feed conversion ratio, and ultimate body weight. No significant effect on diarrhea incidence and mortality rate.	

## Data Availability

No new data were created or analyzed in this study.

## References

[1] Carabaño R., Piquer J., Menoyo D., Badiola I., de Blas C., Wiseman J. (2010). The Digestive System of the Rabbit. Nutrition of the Rabbit.

[2] Gidenne T., García J., Lebas F., Licois D., De Blas C., Wiseman J. (2010). Nutrition and Feeding Strategy: Interactions with Pathology. Nutrition of the Rabbit.

[3] Davies R.R., Davies R.J.A.E. (2003). Rabbit Gastrointestinal Physiology. Vet. Clin. Exot. Anim. Pract..

[4] Bäuerl C., Collado M.C., Zúñiga M., Blas E., Pérez Martínez G. (2014). Changes in Cecal Microbiota and Mucosal Gene Expression Revealed New Aspects of Epizootic Rabbit Enteropathy. PLoS ONE.

[5] Viliene V., Slausgalvis V., Raceviciute-Stupeliene A., Sasyte V., Al-Saifi J., Pockevicius A., Damasaite D. (2018). The Effect of Organic Acids, Medium Chain Fatty Acids and Yeast Cell Walls on Productivity, Physiological State and Intestinal Histomorphology of Rabbits. Vet. Zootech..

[6] Ni M., Wang Z., Li Z., Chen M., He H., Cai H., Chen Z., Li M., Xu H. (2025). Dietary Supplement of Sodium Butyrate Improves the Growth Performance and Intestinal Health by Targeting Wnt/β-Catenin Signaling Pathway in Rabbits. Anim. Res. One Health.

[7] Pearlin B.V., Muthuvel S., Govidasamy P., Villavan M., Alagawany M., Farag M.R., Dhama K., Gopi M. (2020). Role of Acidifiers in Livestock Nutrition and Health: A Review. J. Anim. Physiol. Anim. Nutr..

[8] Khan R.U., Naz S., Raziq F., Qudratullah Q., Khan N.A., Laudadio V., Tufarelli V., Ragni M. (2022). Prospects of Organic Acids as Safe Alternative to Antibiotics in Broiler Chickens Diet. Environ. Sci. Pollut. Res..

[9] Sánchez B., Champomier-Vergès M.C., Collado M.D.C., Anglade P., Baraige F., Sanz Y., De Los Reyes-Gavilán C.G., Margolles A., Monique Z. (2007). Low-PH Adaptation and the Acid Tolerance Response of *Bifidobacterium longum* Biotype Longum. Appl. Environ. Microbiol..

[10] Nguyen D.H., Seok W.J., Kim I.H. (2020). Organic Acids Mixture as a Dietary Additive for Pigs—A Review. Animals.

[11] Elbaz A.M., Farrag B., Mesalam N.M., Basuony H.A., Badran A.M.M., Abdel-Moneim A.M.E. (2023). Growth Performance, Digestive Function, Thyroid Activity, and Immunity of Growing Rabbits Fed Olive Cake with or without Saccharomyces Cerevisiae or Citric Acid. Trop. Anim. Health Prod..

[12] Maklad E., Ragab M., Shalaby N., Ead H., Ragb M. (2023). Effect of Citric Acid Supplementation on Nutrients Digestibility, Nutritive Values and Intestinal Histomorphology of Growing Rabbits. J. Anim. Poult. Prod..

[13] Ramón-Moragues A., Vaggi C.M., Franch-Dasí J., Martínez-Paredes E., Peixoto-Gonçalves C., Ródenas L., del Carmen López-Luján M., Marín-García P.J., Blas E., Pascual J.J. (2024). Screening of Organic Acid Type and Dosage in Drinking Water for Young Rabbits. Animals.

[14] Lin Z., Yang G., Zhang M., Yang R., Wang Y., Guo P., Zhang J., Wang C., Liu Q., Gao Y. (2023). Dietary Supplementation of Mixed Organic Acids Improves Growth Performance, Immunity, and Antioxidant Capacity and Maintains the Intestinal Barrier of Ira Rabbits. Animals.

[15] Ni M., He H., Chen M., Li Z., Cai H., Chen Z., Li M., Xu H. (2024). Supplementation of Sodium Acetate Improves the Growth Performance and Intestinal Health of Rabbits through Wnt/β-Catenin Signaling Pathway. J. Anim. Sci..

[16] Li C., Chen X., Zhang B., Liu L., Li F. (2020). Sodium Butyrate Improved Intestinal Barrier in Rabbits. Ital. J. Anim. Sci..

[17] Zhu K.H., Xu X.R., Sun D.F., Tang J.L., Zhang Y.K. (2014). Effects of Drinking Water Acidification by Organic Acidifier on Growth Performance, Digestive Enzyme Activity and Caecal Bacteria in Growing Rabbits. Anim. Feed Sci. Technol..

[18] Gidenne T., Combes S., Fortun-Lamothe L. (2012). Feed Intake Limitation Strategies for the Growing Rabbit: Effect on Feeding Behaviour, Welfare, Performance, Digestive Physiology and Health: A Review. Animal.

[19] Wang J., Wu Y., Zhou T., Feng Y., Li L.A. (2025). Common Factors and Nutrients Affecting Intestinal Villus Height—A Review. Anim. Biosci..

[20] Hassanin A., Tony M.A., Sawiress F.A.R., Abdl-Rahman M.A., Saleh S.Y. (2015). Influence of Dietary Supplementation of Coated Sodium Butyrate and/or Synbiotic on Growth Performances, Caecal Fermentation, Intestinal Morphometry and Metabolic Profile of Growing Rabbits. J. Agric. Sci..

[21] Kishawy A.T.Y., Amer S.A., Osman A., Elsayed S.A.M., Abd El-Hack M.E., Swelum A.A., Ba-Awadh H., Saadeldin I.M. (2018). Impacts of Supplementing Growing Rabbit Diets with Whey Powder and Citric Acid on Growth Performance, Nutrient Digestibility, Meat and Bone Analysis, and Gut Health. AMB Express.

[22] Romero C., Rebollar P.G., DaI Bosco A., Castellini C., Cardinali R. (2011). Dietary Effect of Short-Chain Organic Acids on Growth Performance and Development of Intestinal Lymphoid Tissues in Fattening Restricted Rabbits. World Rabbit Sci..

[23] den Besten G., van Eunen K., Groen A.K., Venema K., Reijngoud D.J., Bakker B.M. (2013). The role of short-chain fatty acids in the interplay between diet, gut microbiota, and host energy metabolism. J. Lipid Res..

[24] Kim J.W., Kim J.H., Kil D.Y. (2015). Dietary Organic Acids for Broiler Chickens: A Review. Rev. Colomb. Cienc. Pecu..

[25] Klisevičiute V., Slausgalvis V., Racevičiute-Stupeliene A., Sasyte V., Gružauskas R., Al-Saifi J., Pankinaite S., Pockevičius A. (2016). Influence of Dietary Inclusion of Butyric Acid and Organic Acid Salt Mixture on Rabbits’ Growth Performance and Development of Digestive Tract. Vet. Zootech..

[26] Guilloteau P., Martin L., Eeckhaut V., Ducatelle R., Zabielski R., Van Immerseel F. (2010). From the Gut to the Peripheral Tissues: The Multiple Effects of Butyrate. Nutr. Res. Rev..

[27] Emami K.N., Daneshmand A., Zafari Naeini S., Graystone E.N., Broom L.J. (2017). Effects of Commercial Organic Acid Blends on Male Broilers Challenged with *E. coli* K88: Performance, Microbiology, Intestinal Morphology, and Immune Response. Poult. Sci..

[28] Debi M., Islam K., Akbar M., Ullha B., Das S. (2010). Response of Growing Rabbits to Different Levels of Dietary Citric Acid. Bangladesh J. Anim. Sci..

[29] Uddin M., Islam K., Reza A., Chowdhury R. (2014). Citric Acid as Feed Additive in Diet of Rabbit-Effect on Growth Performance. J. Bangladesh Agric. Univ..

[30] Zeweil H.S., Ahmed M.H., Basyony M. (2010). Productive Performance, Carcass Traits and Some Physiological Changes in Rabbits Fed on Diets Containing Acacia Desert Plants and Supplemented with Organic Acids. Egypt. J. Rabbit Sci..

[31] Ismail I.E., Bealish A.M.A. (2014). Effect of Acetic Acid Addition on Growth and Some Physiological and Immunological Characteristics of Growing Rabbits. Egypt. J. Rabbit Sci..

[32] Afroze S., Meng F., Jensen K., McDaniel K., Rahal K., Onori P., Gaudio E., Alpini G., Glaser S.S. (2013). The Physiological Roles of Secretin and Its Receptor. Ann. Transl. Med..

[33] Chowdhury R., Rahman M., Al Mamun M. (2022). Influence of Dietary Organic Acid, Probiotic and Antioxidant on the Growth Performance and Nutrient Digestibility in Growing Rabbit. Bangladesh J. Anim. Sci..

[34] Li D., Li C., Liu N., Liu H., Yu Z., Liu Q., Shu G., Lin J., Zhang W., Peng G. (2025). Integrated Metabolomics and Intestinal Microbiota Analysis to Reveal Anti-Post-Weaning Diarrhea Mechanisms of Modified Yupingfeng Granule in Rex Rabbits. Front. Microbiol..

[35] Skřivanová E., Marounek M. (2007). Influence of PH on Antimicrobial Activity of Organic Acids against Rabbit Enteropathogenic Strain of *Escherichia coli*. Folia Microbiol..

[36] Chukwudi P., Umeugokwe P.I., Ikeh N.E., Amaefule B.C. (2025). The Effects of Organic Acids on Broiler Chicken Nutrition: A Review. Anim. Res. One Health.

[37] Kundu S., Das S., Maitra P., Halder P., Koley H., Mukhopadhyay A.K., Miyoshi S., Dutta S., Chatterjee N.S., Bhattacharya S. (2025). Sodium Butyrate Inhibits the Expression of Virulence Factors in Vibrio Cholerae by Targeting ToxT Protein. mSphere.

[38] Ebeid T.A., Zeweil H.S., Basyony M.M., Dosoky W.M., Badry H. (2013). Fortification of Rabbit Diets with Vitamin E or Selenium Affects Growth Performance, Lipid Peroxidation, Oxidative Status and Immune Response in Growing Rabbits. Livest. Sci..

[39] Chen W., Ma Q., Li Y., Wei L., Zhang Z., Khan A., Khan M.Z., Wang C. (2025). Butyrate Supplementation Improves Intestinal Health and Growth Performance in Livestock: A Review. Biomolecules.

[40] Al-Homidan I., Fathi M., Abdelsalam M., Ebeid T., Abou-Emera O., Mostafa M., El-Razik M.A., Shehab-El-Deen M. (2022). Effect of Propolis Supplementation and Breed on Growth Performance, Immunity, Blood Parameters and Cecal Microbiota in Growing Rabbits. Anim. Biosci..

[41] Hoyles L., Snelling T., Umlai U.K., Nicholson J.K., Carding S.R., Glen R.C., McArthur S. (2018). Microbiome–Host Systems Interactions: Protective Effects of Propionate upon the Blood–Brain Barrier. Microbiome.

[42] Jiang W., Tang P., Lyu S., Brusseau M.L., Xue Y., Zhang X., Qiu Z., Sui Q. (2019). Enhanced Redox Degradation of Chlorinated Hydrocarbons by the Fe(II)-Catalyzed Calcium Peroxide System in the Presence of Formic Acid and Citric Acid. J. Hazard. Mater..

[43] Liang Z.-L., Chen F., Park S., Balasubramanian B., Liu W.-C. (2022). Impacts of Heat Stress on Rabbit Immune Function, Endocrine, Blood Biochemical Changes, Antioxidant Capacity and Production Performance, and the Potential Mitigation Strategies of Nutritional Intervention. Front. Vet. Sci..

[44] Haines R.A., Urbiztondo R.A., Haynes R.A.H., Simpson E., Niewiesk S., Lairmore M.D. (2016). Characterization of New Zealand White Rabbit Gut-Associated Lymphoid Tissues and Use as Viral Oncology Animal Model. ILAR J..

[45] Tian M., Li L., Tian Z., Zhao H., Chen F., Guan W., Zhang S. (2022). Glyceryl Butyrate Attenuates Enterotoxigenic *Escherichia coli*-Induced Intestinal Inflammation in Piglets by Inhibiting the NF-ΚB/MAPK Pathways and Modulating the Gut Microbiota. Food Funct..

[46] Sikandar A., Zaneb H., Younus M., Masood S., Aslam A., Khattak F., Ashraf S., Yousaf M.S., Rehman H. (2017). Effect of Sodium Butyrate on Performance, Immune Status, Microarchitecture of Small Intestinal Mucosa and Lymphoid Organs in Broiler Chickens. Asian-Australas. J. Anim. Sci..

[47] Yang X., Xin H., Yang C., Yang X. (2018). Impact of Essential Oils and Organic Acids on the Growth Performance, Digestive Functions and Immunity of Broiler Chickens. Anim. Nutr..

[48] Nguyen D.H., Kim I.H. (2020). Protected Organic Acids Improved Growth Performance, Nutrient Digestibility, and Decreased Gas Emission in Broilers. Animals.

[49] Scapinello C., Garcia de Faria H., Furlan A.C., Michelan A.C. (2001). Effect of the Use of Mannan Oligosaccharides and Acidifiers on the Performance of Growing Rabbits. Rev. Bras. Zootec..

[50] Michelan A.C., Scapinello C., Natali M.R.M., Furlan A.C., Sakaguti E.S., Faria H.G., Santolin M.L.R., Hernandes A.B. (2002). Utilization of Probiotics, Organic Acids and Antibiotics in Diets for Growing Rabbits: Digestibility, Intestinal Morphometry and Performance Evaluation. Rev. Bras. Zootec..

[51] Hollister A.G., Cheeke P.R., Robinson K.L., Patton N.M. (1990). Effects of Dietary Probiotics and Acidifiers on Performance of Weaning Rabbits. J. Appl. Rabbit Res..

[52] Maertens L., Falcão-e-Cunha L., Marounek M., Maertens L., Coudert P. (2006). Feed Additives to Reduce the Use of Antibiotics. Recent Advances in Rabbit Science.

[53] Skřivanová V., Marounek M. (2002). Effect of Caprylic Acid on Performance and Mortality of Growing Rabbits. Vet. Med. Czech.

[54] Skřivanová V., Marounek M. (2006). A Note on the Effect of Triacylglycerols of Caprylic and Capric Acid on Performance, Mortality, and Digestibility of Nutrients in Young Rabbits. Anim. Feed Sci. Technol..

[55] Ebeid T.A., Al-Homidan I.H. (2022). Organic Acids and Their Potential Role for Modulating the Gastrointestinal Tract, Antioxidative Status, Immune Response, and Performance in Poultry. Worlds Poult. Sci. J..

